# Cardiac autonomic modulation induced by doxorubicin in a rodent model of colorectal cancer and the influence of fullerenol pretreatment

**DOI:** 10.1371/journal.pone.0181632

**Published:** 2017-07-20

**Authors:** Nejka Potočnik, Martina Perše, Anton Cerar, Rade Injac, Žarko Finderle

**Affiliations:** 1 Institute of Physiology, Faculty of Medicine, University of Ljubljana, Ljubljana, Slovenia; 2 Institute of Pathology, Medical Experimental Centre, Faculty of Medicine, University of Ljubljana, Ljubljana, Slovenia; 3 Department of Pharmaceutical Biology, Faculty of Pharmacy, University of Ljubljana, Ljubljana, Slovenia; Virginia Commonwealth University, UNITED STATES

## Abstract

The very effective anticancer drug doxorubicin (DOX) is known to have cardiotoxic side effects, which could be accompanied by autonomic modulation. Autonomic disbalance might even be an initiating mechanism underlying DOX-induced cardiotoxicity and can be studied noninvasively by the analysis of heart rate variability (HRV). A number of strategies have been assessed to predict chemotherapy-induced cardiac dysfunction while HRV, a potential detecting tool, has not yet been tested. Thus, we aimed to determine the effect of DOX treatment on HRV in a rat model of colorectal cancer. While pretreatment with fullerenol (Frl) acts protectively on DOX-induced cardiotoxicity, we aimed to test the effect of Frl pretreatment on DOX-induced HRV alterations. After the induction of colorectal cancer, adult male Wistar rats were treated with saline (n = 7), DOX (1.5 mg/kg per week, n = 7) or DOX after pretreatment with Frl (25 mg/kg per week, n = 7) for three weeks (cumulative DOX dose 4.5 mg/kg). One week after treatment rats were anaesthetized, standard ECG was measured and HRV was analyzed in time and frequency domain. During autopsy the intestines and hearts were gathered for biochemical analysis and histopathological examination. DOX treatment significantly decreased parasympathetically mediated high-frequency component (p<0.05) and increased the low-frequency component of HRV (p<0.05), resulting in an increased LF/HF ratio (p<0.05) in cancerous rats. When pretreated with Frl, DOX-induced HRV alterations were prevented: the high-frequency component of HRV increased (p<0.01), the low-frequency decreased (p<0.01), LF/HF ratio decreased consequently (p<0.01) compared to DOX only treatment. In all DOX-treated animals, disbalance of oxidative status in heart tissue and early myocardial lesions were found and were significantly reduced in rats receiving Frl pretreatment. Autonomic modulation accompanied the development of DOX-induced cardiotoxicity in rat model of colorectal cancer and was prevented by Frl pretreatment. Our results demonstrated the positive prognostic power of HRV for the early detection of DOX-induced cardiotoxicity.

## Introduction

Doxorubicin (DOX) is an important effective drug for the treatment of cancer patients. However, its use is limited by acute and chronic side effects, such as hepatotoxicity, nephrotoxicity, pulmotoxicity and cardiotoxicity [1–3]. The cardiotoxicity is characterized by electrophysiological, biochemical and morphological alterations [4] leading to irreversible cardiac dysfunction [1] and to the development of overt heart failure [5]. Suggested contributors to DOX-induced cardiomyopathy include mitochondrial disruption [6], inadequate cellular energetic [7], formation of free radicals [8–9], apoptosis [10], inhibited expression of cardiomyocyte-specific genes [10], production of proinflammatory, reduction of antiinflammatory cytokines [11] and alteration of adrenergic system in the heart [1,12], but the patophysiology is still not completely understood [1, 6–10]. It is commonly accepted that DOX increases oxidative stress [9], which could be particularly harmful to cardiomyocytes with sparse antioxidant defense [13]. Thus, the use of antioxidants as protective agents could have beneficial effects on DOX-induced toxicity, especially on cardiotoxicity. A lot of studies found a protective effect of fullerenol C_60_(OH)_24_ (Frl) as a potent free radical scavenger in biological systems [2,14,15] and confirmed its role in the prevention of acute DOX-induced cardiotoxicity [3,4]. No effect of Frl treatment on DOX anticancer activity was reported [3]. Our previously published paper [3] reported the lengthening of QRS and SαT segments of ECG in rats treated with DOX after tumor induction and the normal ECG pattern in rats pretreated with Frl.

Many studies suggest that neurohumoral and autonomic tone imbalance play an important role in the pathophysiology of heart disease in humans and in various animal models [16–20]. A powerful noninvasive tool that allows studying the autonomic cardiovascular modulation is the analysis of heart rate variability (HRV) [21–23]. The reduction in HRV is strongly related to the severity of heart disease [24,25] and could have a reliable prognostic value for its clinical outcome [24–29]. Furthermore, the study of DeAngelis et al. [30] reported that alterations in autonomic modulation may be an initiating mechanism underlying the onset of cardiovascular diseases. The results of the study of Lončar-Turukalo et al. [31] confirmed HRV alteration as an early marker of cardiotoxicity in DOX-induced cardiomyopathy in healthy rats. Since it is known that illness itself can provoke autonomic modulation [32], a rat model of colorectal cancer was applied in our study to reveal whether HRV changes can be used as a marker of DOX-induced cardiotoxicity in cancer.

Because DOX-induced cardiotoxicity can cause irreversible damage to the myocardium, prevention is the most effective strategy. It is crucial to detect subclinical signs of cardiotoxicity before cardiac dysfunction develops. Since dose-dependent DOX-induced damage to the tissue was found in dogs [33] and the cumulative dose of DOX was reported to be the most important risk factor for the development of cardiotoxicity in humans [34], it is crucial to find a strategy to detect early signs of cardiotoxicity occurring at a low cumulative DOX dose.

To the best of our knowledge, there are no available studies on the impact of DOX and DOX applied after pretreatment with Frl, a potent drug against the DOX cardiotoxicity, on HRV in cancer.

The aim of our study was to determine whether HRV monitoring could be a promising strategy for early detection of DOX-induced cardiotoxicity. Thus, we studied the effect of low cumulative DOX dose treatment on HRV in a rat model of colorectal cancer and the potential beneficial effect of Frl pretreatment on DOX-induced HRV alteration. In order to explore the effect of DOX and Frl on the cardiac function of the rats with colon cancer, lactate dehydrogenase (LDH) activity, antioxidant defense system capabilities and histological examination of the rat hearts were assessed simultaneously.

## Materials and methods

### Animals

In an experiment 21 male Wistar rats (HsdRccHan^TM^:WIST) were used at 7 weeks of age (bred and maintained at the Medical Experimental Centre, Slovenia). They were quarantined and housed 3–5 per cage (Ehret, Germany; 1825 cm^2^ floor space) on Lignocel ¾ bedding material (Germany) in a standard controlled environmental with conditions at 22±1°C room temperature, 55 ± 10% humidity and a 12 h light/dark cycle (illumination between 07.00 p.m. and 07.00 a.m.). They had free access to a standard laboratory diet (Altromin 1324, Germany) and tap water. Clinical state and body weights of rats were monitored on a weekly basis. The experiment was approved by the Ethical Committee for experiments on animals of the Republic of Slovenia and the Veterinary Administration of the Republic of Slovenia (Permit No. 34401-61/2007/7) and was conducted in accordance with the European Convention for the protection of vertebrate animals used for experimental purposes. Colon cancer was induced by 1,2-dimethylhydrazine (DMH) (Fluka Chemie, Switzerland), prepared according to the standard method [35]. All rats received subcutaneous applications of DMH (20 mg/kg) once a week during 15 weeks. The animals were randomly divided into three groups (7 per group): control group, DOX group and Frl/DOX group. At 24th, 25th and 26th week of age, rats were treated with saline only (control group), with 1.5 mg DOX/kg per week [36,37] (DOX group) and with DOX at the same dose as the DOX group but pretreated with 25 mg Frl/kg per week [3] 30 minutes before DOX (Frl/DOX group).

### Measurements

Seven days after the last application the rats were anesthetized with ketamine (75 mg/kg IP) and xylazine (9 mg/kg IP). A standard 6 channel ECG (Cardiax, IMEDE, Budapest, Hungary) was measured for 3 minutes using subcutaneous needle electrodes. All recordings were carried out between 10 and 10.30 AM. The data were stored in a computer database using Cardiosoft ECG software (Huston, Texas, USA). After ECG measurement the animals underwent further investigations and were sacrificed at the end. The survival rate of the animals was 100%.

### Histology

At autopsy all internal organs were removed, weighted and macroscopically examined. The intestine was opened longitudinally, flushed with tap water, pinned on cardboard, and examined macroscopically for the presence of tumors. The location, number and size of the tumors were recorded and the intestine, heart and all detected lesions were fixed in 4% buffered formalin.

Large intestine was cut longitudinally and sent for histological examination in total length along with all the macroscopically visible lesions. All tissue samples of the large intestine were embedded in paraffin, serially sectioned at 4–5μm and stained with Kreyberg-Jareg method. Aberrant crypts (AC) with dysplastic epithelium, adenomas and carcinomas were assessed by histological criteria described elsewhere [38].

Each heart was quickly removed from the sacrificed rat, placed in an ice-cold solution and divided into two parts. One part taken for histology was fixed in 10% buffered formalin, embedded in paraffin and stained with haematoxylin-eosin. The rat hearts were scored individually for the following parameters: the intensity and diffusion of cytoplasmic myofibrillar loss, cytoplasmic vacuolation, apoptosis, necrosis and degeneration of cardiomyocytes, edema, congestion and central vein thrombosis, and infiltration of poly- or mononuclear cells in the heart sections. [3]. Scoring was done as follows: (0) none, (1) focal-mild, (2) multifocal-moderate and (3) widespread-progressive, respectively. Histological evaluation of tissue samples was carried out blindly.

### Biochemical analysis

LDH activity in the heart tissue was assayed for evaluating cardiac damage [39]. The oxidative status of the heart tissue was assessed by determining the levels of oxidative stress markers (malondialdehyde (MDA), glutathione (GSH), glutathione disulfide (GSSG)) and oxidative enzyme activities [superoxide dismutase (SOD) and catalase (CAT)].

The second part of the heart was minced and homogenized in a Tris-buffer solution (pH 7.4; organ: buffer 1:10; w/w) and divided into two portions; one was used for MDA determination (Chromsystems Diagnostic, Munchen, Germany), and the other was centrifuged at 13,000 × g for 20 min at 4°C (Beckman refrigerated, Ultracentrifuge). The supernatant was used for the assays of LDH (Chema Diagnostica, Jesi, Italy), GSH and GSSG (Chromsystems Diagnostic, Munchen, Germany), SOD (Ransod, Crumlin, UK) and CAT [2]. Free GSH/GSSG ratio was calculated.

### Data processing and statistical analysis

The R peaks of ECG were detected using Nevrokard software (SA aHRV file preparation, Medistar, Slovenia); the eventual R peak detection artifacts were removed manually, R-R interval (RR) time series was obtained. To analyze the spontaneous fluctuations in RR interval in time and frequency domain, advanced heart rate variability analysis for small animals (SA aHRV, Nevrokard, Medistar, Slovenia) was applied.

In the time domain HRV was determined as the standard deviation of successive normal RRs, measured between consecutive sinus beats (SDNN) and as the root mean square of successive differences between normal RRs (RMSSD).

Spectral analysis of RR variability was calculated by fast Fourier transform algorithm using Welch’s method with a Hanning window (256) and 50% overlapping in accordance with the standard spectral estimation procedures [40].

Usually, three oscillatory components are distinguished in the spectral profile: the very low (VLF), the low (LF), and the high-frequency (HF) band [40]. The VLF was not evaluated in our study because the ECG measurement was too short to give reliable results in VLF band [40]. Power spectrum analysis was evaluated by LF power in the frequency range 0.20–0.75 Hz [41], which is related to the interplay between cardiac sympathetic and parasympathetic influences [40] and HF power in the 0.75–2.5 Hz [41] frequency band, which is exclusively under parasympathetic (vagal) control [23,40,41]. The peak frequency in the HF domain corresponds to the respiratory sinus arrhythmia [40]. Each of both power spectral components was calculated as integrals under the respective part of the power spectral density function and expressed in normalized units (nLF and nHF) according to the standard procedure [40,42]. Normalized LF power of HRV was proposed to reflect sympathetic cardiac modulation [40], particularly when the cardiac-sympathetic drive is activated [43]. The LF/HF ratio was calculated and considered as an index of sympathovagal balance [42,44]_._

All results are presented as mean ± standard error of means. Statistical comparison between experimental groups was done using One way ANOVA procedure in Sigma Stat 2.3 software for Windows. LSD post-hoc test was applied for pairwise comparison of histological data and Bonferonni t-test for HRV data. The significance level was set at p < 0.05.

## Results

### Health status of rats

**Fig 1:**
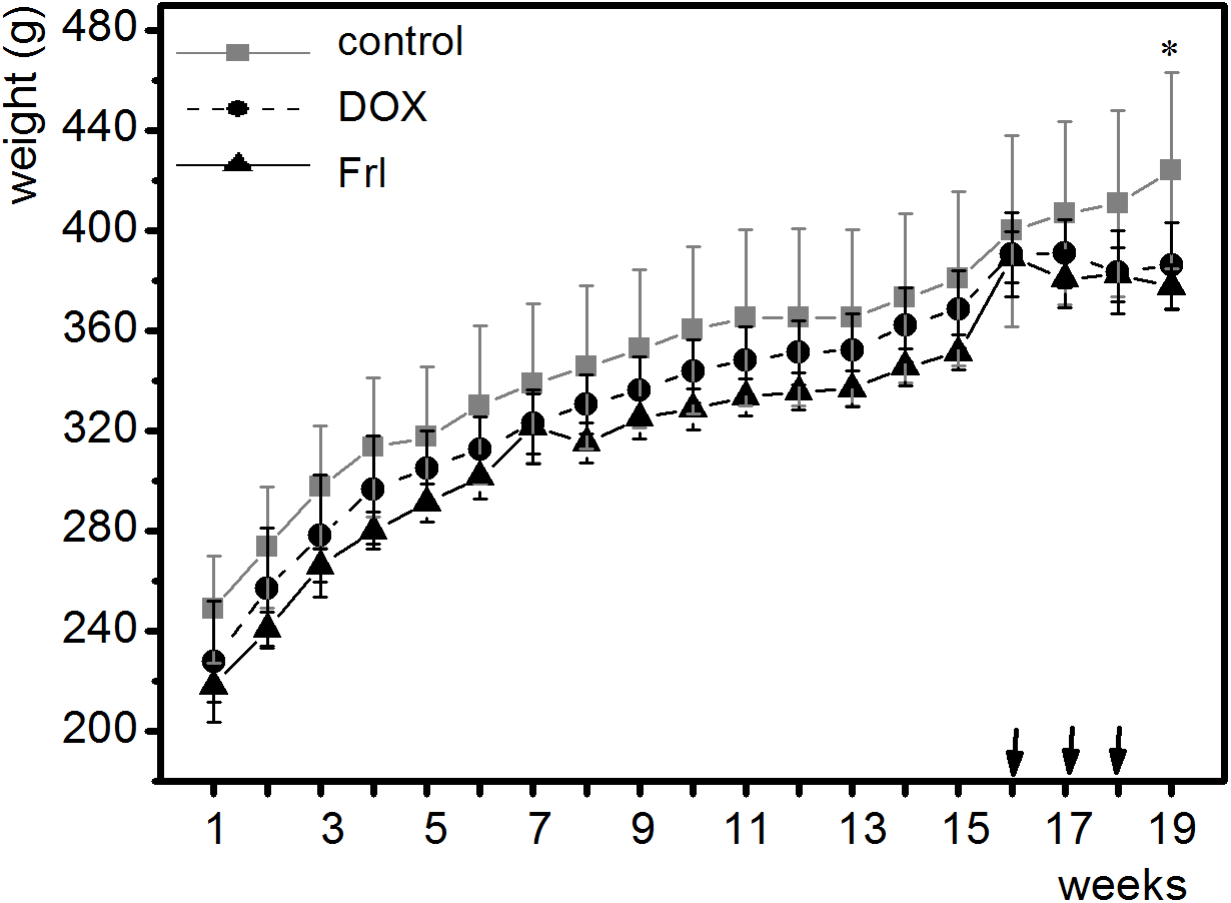
Body weight of rats throughout the experiment. Each curve represents one group of animals. Control: group of rats with colon cancer without anticancer treatment; DOX: doxorubicin-treated group; Frl/DOX: doxorubicin-treated group with fullerenol pretreatment. *:*p* < 0.05 weight gain at 19th week compared to 16th week in control group, no significant weight gain in the same time interval in both DOX group. Arrows indicate time of Dox administration.

The rats were in good general health status and their body weights increased during experiment till the first Dox administration (Fig 1) showing the toxic adverse effect of Dox therapy.

**Fig 2:**
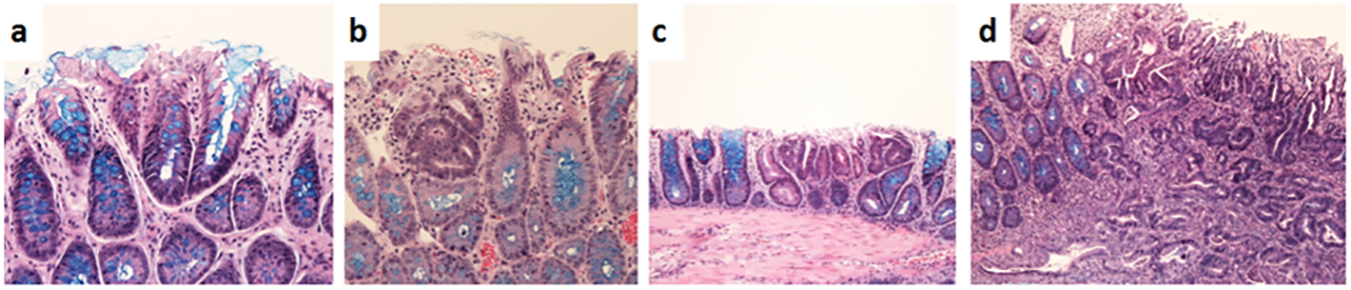
Representative histologic pictures of the colon lesions. All DMH-treated Wistar rats developed small microscopically visible colon lesions such as hyperplastic (a) or dysplastic crypts (b), adenomas (c) and adenocarcinomas (d). Kreyberg staining, magnification 400x (a,b), 200x (c,d).

The histopathological analysis of the intestine revealed that all rats developed small microscopically visible colon lesions (Fig 2). DOX treatment significantly reduced the development of AC and adenocarcinomas in both, Dox (4.1 ± 0.7 ACs; p < 0.05 and 0.6 ± 0.2 adenocarcinomas; p < 0.05) and Frl/DOX (4.1 ± 1.2 ACs; p < 0.05 and 0.4 ± 0.2 adenocarcinomas; p < 0.05) groups compared to controls (9.1 ± 2.2 ACs and 1.4 ± 0.6 adenocarcinomas). The number of adenomas did not differ among groups. Pretreatment with Frl did not affect the DOX anticancer activity.

### Changes in oxidative status and morphology of the heart

DOX resulted in increased oxidative stress in the heart tissue as demonstrated by increased SOD and CAT activities, increased MDA level and decreased GSH/GSSG ratio as well as in increased damage of the heart tissue, demonstrated by increased LDH activities (17.9 ± 3.6 U/L in comparison to both control (6.4 ± 2.8 U/L) and Frl/DOX (3.6 ± 1.8 U/L), p < 0.01). Pretreatment with Frl provided marked normalization of oxidative status: decreased SOD and CAT activities, decreased MDA level and increased GSH/GSSG ratio (p < 0.001) when compared to the group treated with DOX alone.

**Fig 3:**
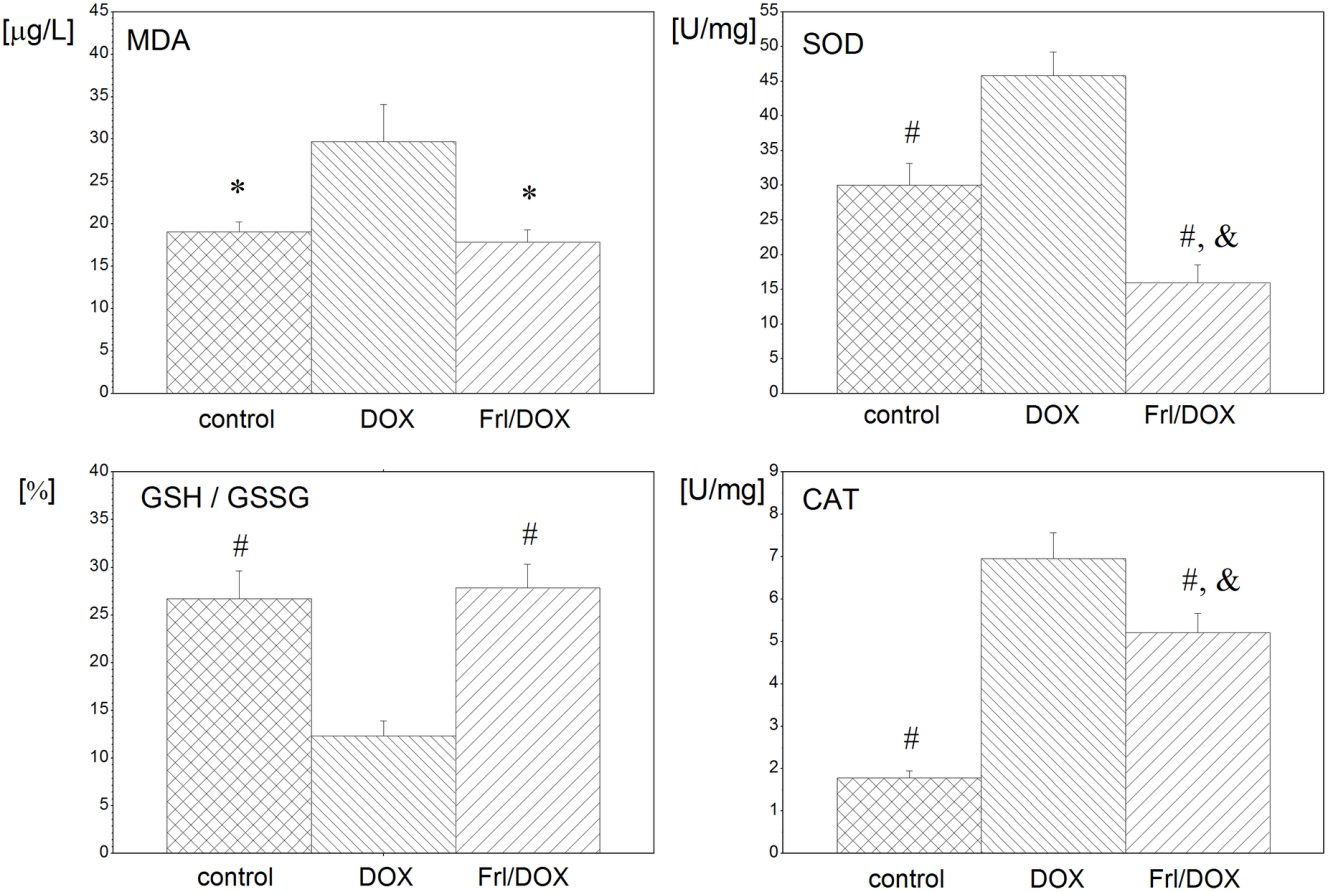
Biochemical evaluation of the heart oxidative status in cancerous rats treated with DOX alone or with Frl pretreatment. MDA: malondialdehyde; GSH/GSSG: free glutathione to free glutathione disulfide ratio; SOD: superoxide dismutase; CAT: catalase. *: p < 0.05 compared to DOX; #: p < 0.01 compared to DOX; &: p < 0.05 compared to control group.

Effects of DOX and Frl treatment on heart oxidative status in rats with colon cancer are summarized in Fig 3.

**Fig 4:**
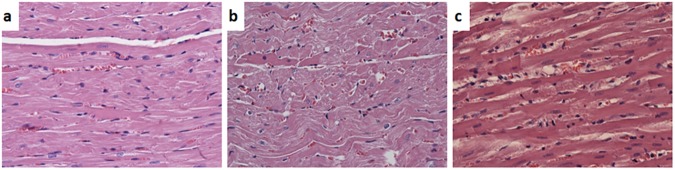
Representative histologic pictures of the heart sections. (a) DMH-treated Wistar rats, control group; (b) after DOX therapy; (c) DOX therapy in combination with Frl pretreatment, haematoxylin-eosin staining, magnification 400x.

Histology confirmed that DOX-induced myocardial lesions, histologically classified as myofibrillar loss, parenchymal degeneration and lymphoid infiltration, were significantly reduced in rats that received Frl pretreatment (Fig 4, Table 1).

**Table 1:** Cardiac lesion scores in cancer controls, rats treated with doxorubicin and doxorubicin pretreated with fullerenol.

	Control	DOX	Frl/DOX
Degeneration	9*	12	9*
Lymphoid infiltration	3*	8	1#
Myofibrillar loss	3#	8	2#

*: p <0.05 compared to DOX

#: p <0.01 compared to DOX.

Significantly better cardiac lesion scores were found in control and Frl/DOX group compared to DOX. Control: group of rats with colon cancer without anticancer treatment; DOX: doxorubicin-treated group; Frl/DOX: doxorubicin-treated group with fullerenol pretreatment.

### Heart rate analysis

**Fig 5:**
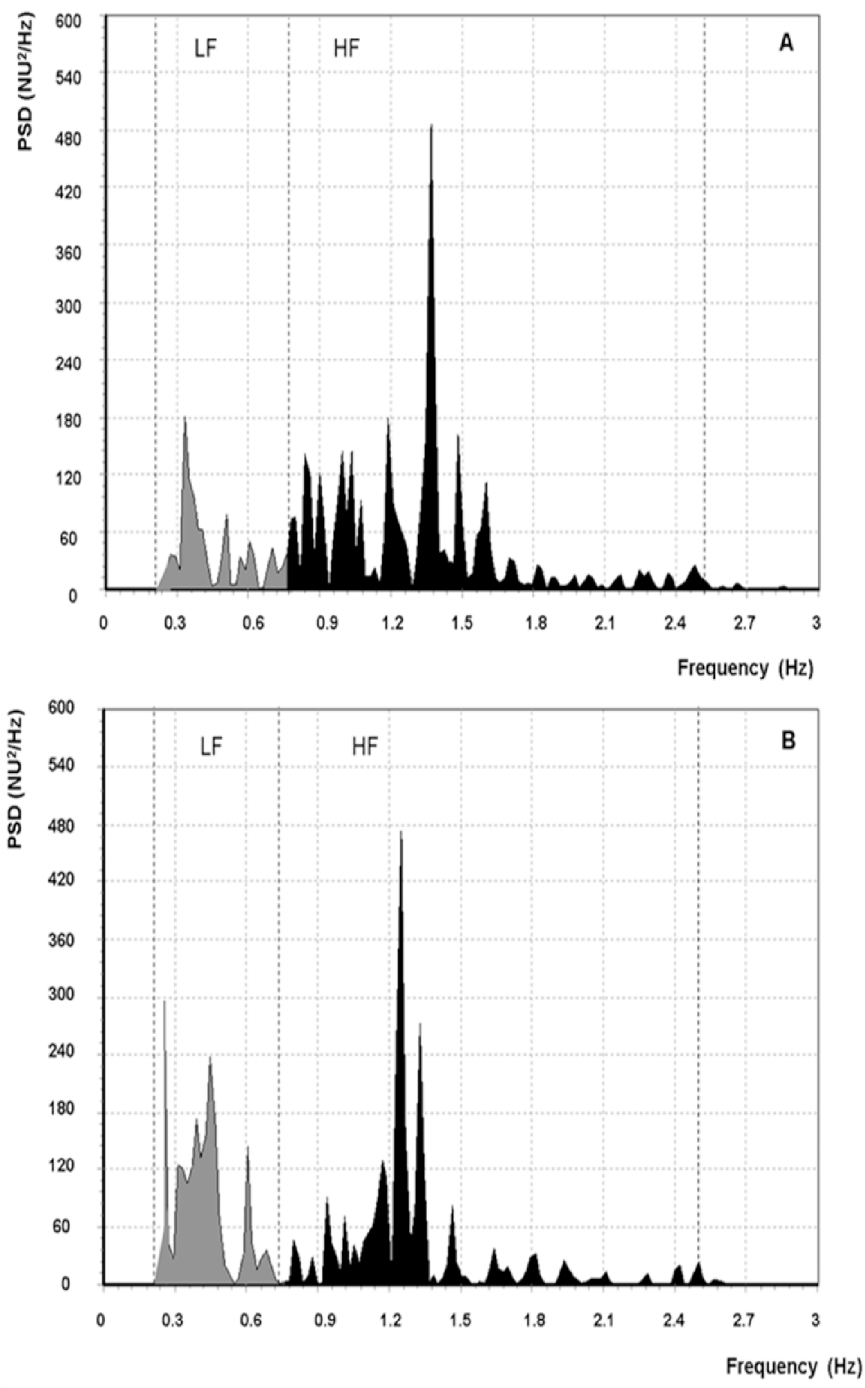
Power spectra of RR intervals for two representative rats. (A) Rat from the control group with colon cancer without anticancer treatment. (B) Rat from the DOX group with colon cancer, treated with doxorubicin (DOX). LF: low-frequency band of power spectrum, HF: high-frequency band of power spectrum, NU: normalized units, PSD: power spectrum density.

DOX administration schedule used in our study in DOX group produced significant changes in the frequency domain of HRV compared to controls. An example of power spectral analysis of HRV in control and DOX-treated animal is given in Fig 5.

**Fig 6:**
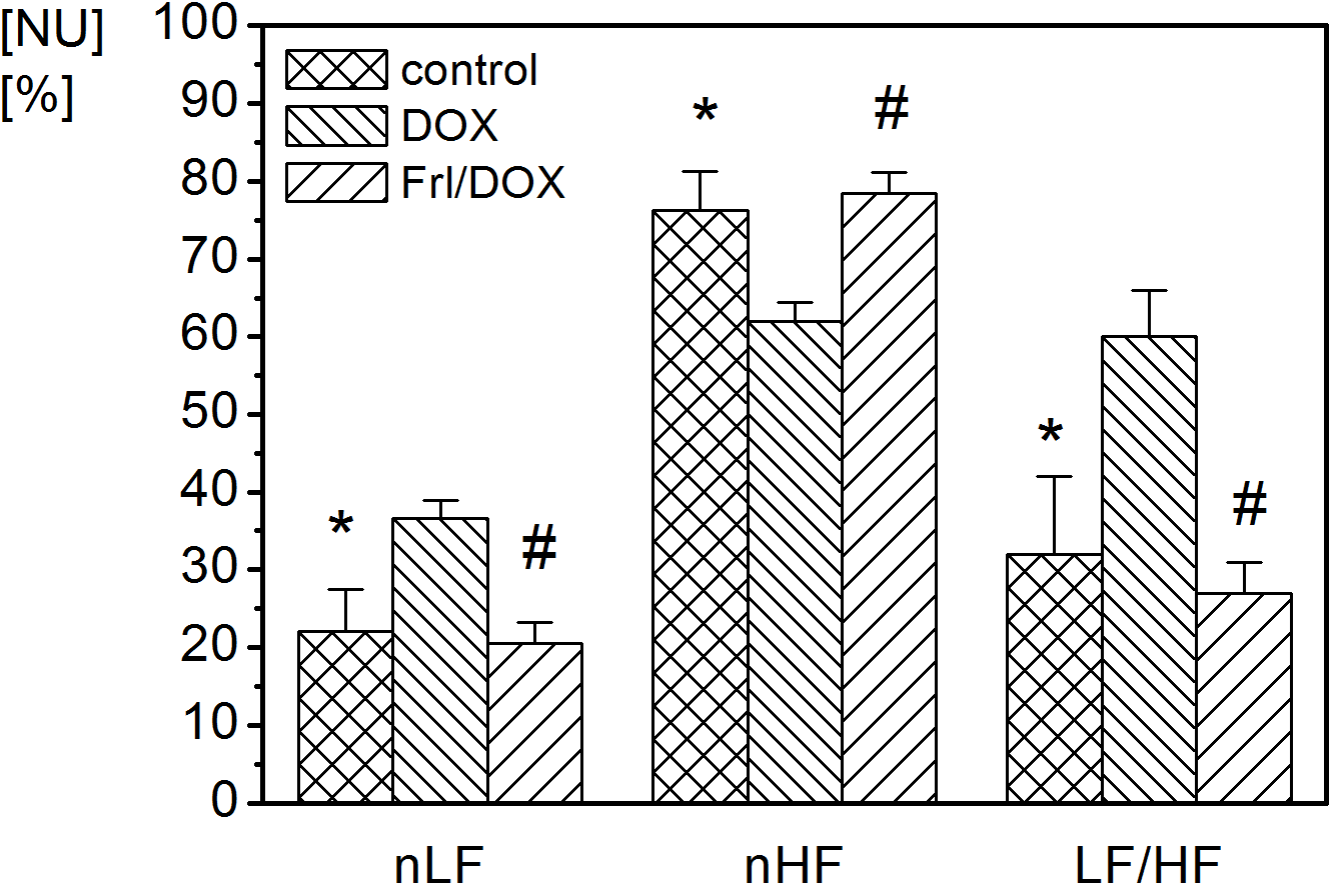
Frequency domain indices in control, DOX and Frl/DOX groups of animals. Statistically significant differences of control and Frl/DOX group were found in nLF, nHF and LF/HF compared to DOX. Control: group of rats with colorectal carcinoma without anticancer treatment, DOX: group of rats with colon cancer, treated with doxorubicin (DOX), Frl/DOX: group of rats with colon cancer, treated with doxorubicin, pretreated with Fullerenol. *—statistically significant difference of control group compared to DOX (p<0.05), #—statistically significant difference of Frl/DOX group compared to DOX (p<0.01). nLF: integral under the low-frequency (LF) part of the power spectral density function expressed in normalized units (NU), nHF: integral under the high frequency (HF) part of the power spectral density function expressed in NU; LF/HF: the ratio between power spectrum density in HF part and LF part of the total power spectrum.

Administration of DOX significantly decreased parasympathetically mediated nHF of HRV (p < 0.05) (Table 2, Fig 6). In contrast, sympathetically mediated nLF of HRV was significantly increased after DOX treatment (p < 0.05), resulting in an overall increased LF/HF ratio (p < 0.05) (Table 2, Fig 6). There were no significant alterations in HRV across both mentioned frequency bands in the Frl/DOX group compared to control group (Table 2, Fig 6). Comparing Frl/DOX to DOX, nHF was increased (p < 0.01) and nLF decreased (p < 0.01) with corresponding LF/HF decrease (p < 0.01) (Table 2, Fig 6).

**Table 2:** Heart rate and heart rate variability parameters in time and frequency domains.

	Control	DOX	Frl/DOX
RR (ms)	234.5±13.7	272.5±17.6	249.6±7.29
HR (min^-1^)	255.8±14.9	220.18±14.2	240.38±7.0
SDNN (ms)	2.8±0.4	5.6±1.4	3.6±0.5
RMSSD (ms)	4.9±0.8	6.6±1.7	6.5±1.5
nLF (NU)	22.13±5.33*	36.65±2.24	20.51±2.68#
nHF (NU)	76.20±5.01*	62.02±2.35	78.50±2.67#
LF/HF	0.32±0.10*	0.60±0.06	0.27±0.04#

*: p <0.05 compared to DOX

#: p <0.01 compared to DOX.

Heart rate variability parameters in frequency domain showed a significant difference in the control group and Frl/DOX compared to DOX. Heart rate is expressed in RR interval duration (RR); SDNN: standard deviation of successive normal RRs, measured between consecutive sinus beats; RMSSD: root mean square of successive differences between normal RRs; nLF: integral under the low-frequency (LF) part of the power spectral density function expressed in normalized units; nHF: integral under the high frequency (HF) part of the power spectral density function expressed in normalized units; LF/HF: the ratio between power spectrum density in HF part and LF part of the total power spectrum. Control: group of rats with induced colon cancer without anticancer treatment; DOX: doxorubicin-treated group; Frl/DOX: doxorubicin-treated group with fullerenol pretreatment.

**Fig 7:**
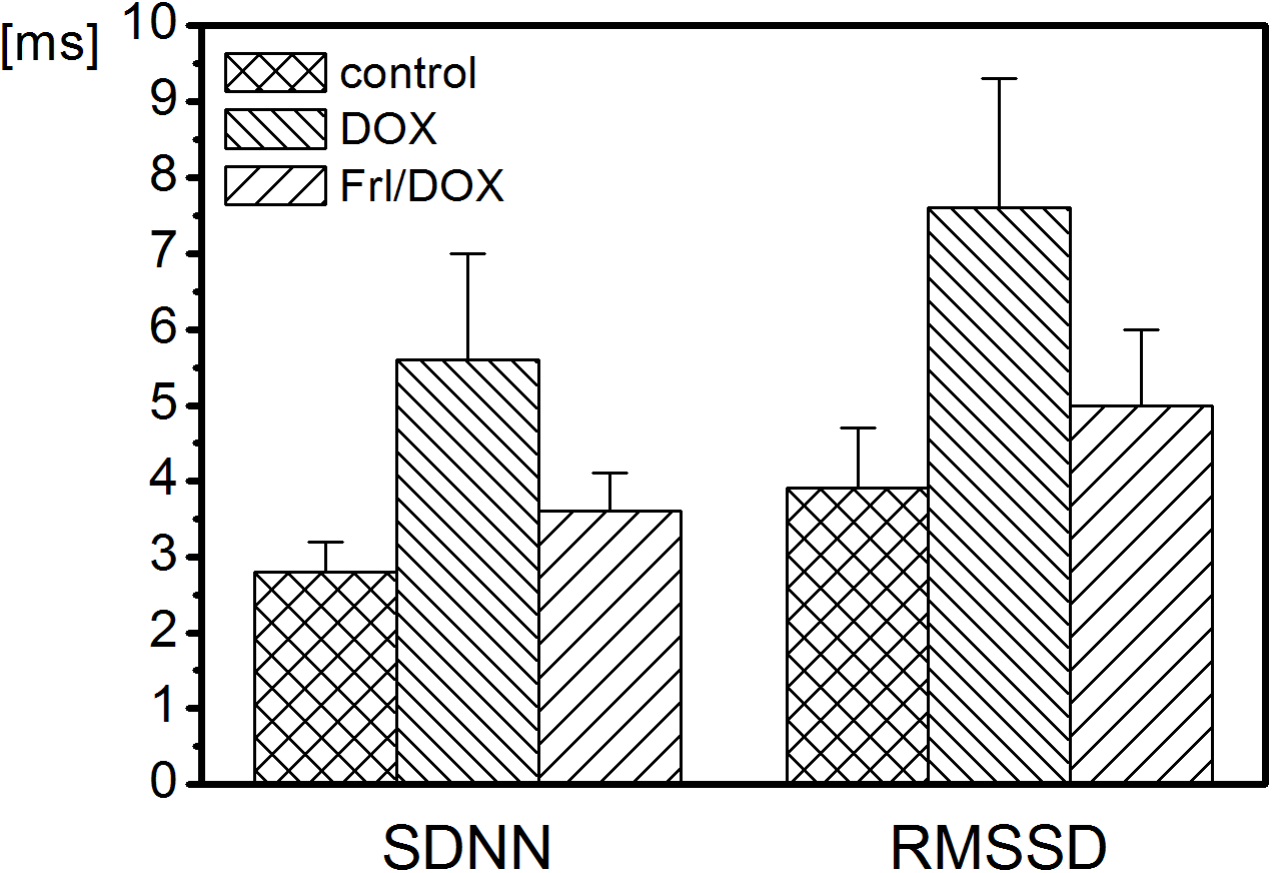
Time domain indices in control, DOX and Frl/DOX groups of animals. No statistically significant differences in time domain indices were found between groups. Control: group of rats with colon cancer without anticancer treatment, DOX: group of rats with colon cancer, treated with doxorubicin (DOX), Frl/DOX: group of rats with colorectal carcinoma, treated with doxorubicin and pretreated with Fullerenol. SDNN: standard deviation of successive normal RRs, measured between consecutive sinus beats; RMSSD: root mean square of successive differences between normal RR intervals in ECG.

There were no statistically significant changes in heart rate and the HRV parameters in the time domain (Table 2, Fig 7) among the groups studied. However, a tendency toward higher values of time domain indices was observed in the DOX group (Table 2, Fig 7).

## Discussion

The main findings of the present study are as follows: 1.) DOX treatment of cancerous rats (cumulative dose 4.5mg/kg) altered HRV compared to untreated cancerous rats one week after DOX treatment cessation; 2.) pretreatment with Frl, an effective antioxidant and a potent protecting agent against DOX-induced cardiotoxicity, completely prevented HRV changes; 3) HRV alterations in DOX-treated animals were accompanied by elevated oxidative stress of the heart tissue and by histological changes typical for DOX-induced cardiotoxicity, whereas no HRV modulations, less oxidative stress and normal myocardial microscopy were observed when pretreated with Frl.

According to the commonly accepted interpretative HRV guidelines [40,45], the results of our experiment showed that DOX treatment of cancerous animals modulates autonomic system activity to the heart. DOX-induced increases in nLF and LF/HF display the overactivation of cardiac sympathetic drive [40,46] in DOX-treated cancerous rats compared to controls. On the other hand, a DOX-induced decrease in nHF indicates concomitant withdrawal of cardiac parasympathetic activity [40,45] in DOX-treated rats compared to controls. Accordingly, sympathetic overactivity in combination with reduced vagal tone to the heart characterizes the main effect of DOX on cardiac autonomic modulation in cancerous rats. Contrary to DOX-only treatment, pretreatment with Frl did not result in any significant changes in the tones of any particular parts of autonomic activity to the heart, suggesting its protective role against cardiac autonomic disbalance caused by DOX.

Cardiac autonomic disbalance observed in the DOX group in our study was accompanied by elevated oxidative stress of the heart tissue and by histological changes typical of DOX-induced cardiotoxicity [3]. In normal healthy conditions, free oxygen radicals are efficiently detoxified by antioxidant enzymes such as SOD and CAT [47]. In the present study, the activities of SOD and CAT were increased in the DOX group compared to both control and Frl/DOX groups, indicating that myocardial tissue tried to detoxify the oxygen-free radicals but insufficiently. Concerning GSH redox cycle, GSH/GSSG maintenance is crucial to keeping cellular redox status [48]. In our study, GSH/GSSG ratio in heart tissue was found to be significantly decreased in the DOX group as compared to control and Frl/DOX, additionally confirming the disbalance in oxidant-antioxidant status in heart tissue in DOX. As a result, lipid peroxidation and oxidative tissue injury are induced by DOX [9,47]. Indeed, MDA, a marker of lipid peroxidation, was significantly increased in the heart tissue of DOX-treated animals in our study, and heart tissue damage was confirmed by histopathological examination.

Histopathological consequences of doxorubicin-induced cardiotoxicity are demonstrated in several studies. Yagmurca et al. [49] investigated the results of cumulative 20 mg/kg doxorubicin treatment in an experimental rat model of severe cardiotoxicity and found disorganization of myocardial histology. Similarly, Chen et al. [50] reported myofibrillar loss and myocyte degeneration in histopathological examination of myocardium by application of cumulative 15 mg/kg doxorubicin treatment in an experimental rat model. Our histopathological results are in compliance with the above-mentioned reports albeit low cumulative DOX dose (4.5 mg/kg): DOX-induced myocardial lesions, histologically classified as areas of myofibrillar loss, single cell necrosis and parenchymal degeneration, as well as moderate lymphoid infiltration of the heart tissue. During the three consecutive applications of DOX at a dose of 1.5 mg/kg/week, it produced a marked increase in the heart LDH level as compared to control and Frl/Dox group, additionally confirming that heart tissue damage was present.

No HRV changes were found in DOX rats that received Frl pretreatment, indicating that Frl prevents DOX-induced autonomic changes to the heart. Concomitantly, in Frl/DOX group, the heart tissue levels of MDA, LDH and GSH/GSSG ratio returned to their baseline values. The activities of the antioxidant enzymes SOD and CAT decreased and myocardial damage was significantly attenuated in Frl/DOX group. A lot of other studies have already confirmed the protective effects of fullerenols (C_60_(OH)_n_) against the cytotoxicity of DOX in animal models, especially Frl [2,3,51]. In DOX-induced citotoxicity Frl with its high antioxidative potential acts as a free radical sponge and/or removes free iron through the formation of the fullerenol-iron complex [51], which further disables the oxidative cell damage induced by DOX. Additionally, Flr could have an anti-inflammatory effect on the organism after treatment with DOX. On the other hand, current knowledge indicates that fullerenols show low or no toxicity [52] and may contribute to the inhibition of tumor growth and the protection of normal cells through their antioxidant properties, as well as to the regulation of expression of genes involved in apoptosis and angiogenesis, and stimulation of the immune response in humans [52]. Accordingly, pretreatment with Frl did not affect the DOX anticancer activity in the current study.

The same changes in HRV as induced by low cumulative DOX dose in our study are characteristic of the initial phases of congestive heart failure in rodents and humans where a significant increase in LF and a decrease in HF were detected and interpreted corresponding to sympathetic overactivity and reduced vagal tone [53–55]. In addition, statistically significant differences in LF/HF, nLF and nHF were found between normal and cardiovascular risk subjects [16]. Sympathetic overactivity early in DOX-induced cardiotoxicity merges with the published evidence that common pathogenesis for heart diseases of different etiologies could be based on a sympathetic homeostatic shift toward higher activity [20,56].

The link between reduced cardiac vagal activity and heart failure development was clinically confirmed in many studies [54,56,57]. In mice, attenuated parasympathetic tone ultimately causes the progression of depressed left ventricular function to heart failure [56,58].

Our results are consistent with the results of the study by Lončar-Turukalo et al. [31] which evaluated the influence of DOX treatment on the autonomic nervous system activity in healthy rats. In accordance with their study, it can be concluded that DOX treatment equally affects the regulation of the autonomic nervous system activity to the heart in healthy as well as in cancerous animals.

Analyses of cardiac autonomic control based on HRV changes do not provide any evidence of the potential mechanisms involved. However, there are several studies demonstrating the impact of different autonomic and non-autonomic factors on HRV, which could be associated with DOX-induced cardiotoxicity. Concerning autonomic factors, modulation of autonomic nervous activity to the heart, adrenoceptor (AR) and muscarinic receptor density [59, 60], their function [46] and AR receptor activated intracellular signaling pathways [46,61] are proposed while suggested non-autonomic factors mainly include inflammation [62,63] and oxidative stress [46,63,64]. Based on the reports of the above-mentioned studies, some potential mechanisms underlying the HRV modulation in DOX-induced cardiotoxicity observed in the present research might be proposed.

According to the study of Hrushesky et al. [65], it is unlikely that alterations in HRV were provoked by DOX-induced autonomic neuropathy. They proposed that the direct effect of DOX on the heart muscle itself caused HRV changes [65].

Indeed, Merlet et al. [66] revealed increased β2-AR expression and overexpression of inhibitory G protein (Gi), particularly its α-2 subunit (Giα-2) in the early phase of DOX-induced cardiomyopathy in rats. The importance of Giα-2 involvement in fine-tuning of β-AR function in DOX-induced cardiotoxicity was additionally approved by the study of specific microRNAs [67], which play potent regulatory roles in both cardiovascular disease and cancer. While direct in vivo experiment examining the role of Gi signaling in the sinoatrial node [68] found the selective loss of nHF in mice with expressed Giα-2 compared to mice with silenced Giα-2 expression, decreased nHF found in DOX-treated animals in our study supports the involvement of Giα-2 in DOX-induced cardiotoxicity. The above-mentioned findings suggested that the new myocardial phenotype in terms of β-ARs densities and G protein signaling was expressed by DOX and this might be the main determinant of the observed HRV modifications [69].

Concerning parasympathetic modulation, reduced nHF in DOX group, detected in our study, could be further interpreted as a consequence of a decreased expression of M2 receptors in cardiomyocytes of DOX-treated rats [70] in accordance with the study of Ribeiro et al. [71], who showed that M2 receptor level was significantly correlated with HF power values in the human heart.

Depression in HF component of HRV reported during systemic administration of endotoxin to rats is associated with increased cytokine production (IL-6) [72]. While DOX treatment increases cardiac IL-6 [73] level, inflammation could be another explanation for reduced nHF in DOX group compared to both other groups in our study.

Chaswal et al. [74] reported that increased oxidative stress influenced cardiac autonomic response. They found enhanced serum MDA level in L-NAME treated rats associated with the same HRV changes as reported in our research: attenuated HF and increased LF/HF ratio. However, the same study revealed that sympathectomy importantly modifies those HRV changes, reflecting a significant role of autonomic tone to the heart in oxidative stress-induced HRV modulatin, which could also not be excluded in our study.

While HRV was found to depend on HR [75], it is debatable whether this procedure can be used to analyze autonomic nerve activity of the heart when HR is changing. In our study, all groups of rats had similar mean HR (Table 2) justifying the use of HRV to evaluate cardiac autonomic tone which showed evident differences among groups at the same mean HR. While mean HR in common opinion represents the net effect of the simultaneous sympathetic and vagal influences on cardiac pacemaker activity [42], equal LF/HF ratio would be expected in all groups of animals. However, LF/HF ratio differed in DOX group compared to control or Frl/DOX, suggesting that vagal and sympathetic influences on cardiac pacemaker cells were not equally processed as receptor number and function are changed and downstream mechanisms are altered as already discussed in previous paragraphs.

In our research, the time domain HRV parameters (SDNN and RMSSD) did not alter as normally expected at sympathetic overactivation, confirming again the possible impact of non-autonomic factors on HRV in DOX.

The current paradigm for cardiotoxicity detection in humans relies primarily upon the assessment of the left ventricular ejection fraction (LVEF) [76]. Multigated acquisition scan (MUGA) and echocardiography are the most studied methods for the measurement of LVEF in humans; echocardiography was applied also in rats [31,37]. As LVEF measurements are very variable and insensitive [77] and the normal heart has a huge recruitable contractile potential [78], it is important to find other markers for the detection of early cardiac damage. Our study confirmed that HRV monitoring could be a potential strategy. While heart must have undergone a considerable damage and myocyte loss to express the decreased LVEF [78], echocardiography was not employed in our study, where only subtle myocardial changes were expected [3]. Even at higher cumulative DOX dose (7.5 mg/kg), no alterations in echocardiographic scoring were reported [37].

Results of animal studies are valuable for understanding changes in heart rate and heart rate variability generated by pharmacological or pathological manipulations but they have limitations. A lot of analogies exist between rats and humans with respect to HRV [46,68]. However, a number of important caveats remain [46], precluding a simple extrapolation of rodent cardiovascular data to humans.

It is well known that anesthesia itself can provoke autonomic modulation. However, the slightest alterations of HR were observed using ketamine and xylazine anesthesia [79]. Ketamine and xylazine were found to increase parasympathetic activity and suppress sympathetic activity in rat [80]. Since decreased parasympathetic and increased sympathetic activity were found in our study, we might assume that the effects of anesthesia were overridden by the HRV modulation provoked by DOX.

## Conclusions

In conclusion, DOX treatment (cumulative dose 4.5 mg/kg) of rats with colorectal cancer induced HRV changes compatible with sympathetic overactivity and reduced vagal tone to the heart. These HRV changes were accompanied by heart tissue damage, increased oxidative stress and histological changes typical for DOX-induced cardiotoxicity. Pretreatment with Frl completely prevented HRV alterations induced by DOX, decreased oxidative stress and normalized myocardial histopathological score. HRV could be used as a noninvasive tool for early detection of DOX-induced cardiotoxicity in cancer.

## Supporting information

S1 Dataset Biochemical analysis results and histological scoring of intestine and heart. (PDF)

S2 Dataset Heart rate variability analysis. (PDF)
