# Supplementary material for: Cardiac autonomic modulation induced by doxorubicin in a rodent model of colorectal cancer and the influence of fullerenol pretreatment
Source: PLoS One. 2017 Jul 20;12(7):e0181632. doi: 10.1371/journal.pone.0181632 (PMC5519181; doi:10.1371/journal.pone.0181632)
Supplement: S1 Dataset — (PDF) [file pone.0181632.s001.pdf]

intestinal lesions

DMH

HC	adenomas	adencarcin
9	1	0
6	0	0
8	1	4
2	0	1
5	0	1
17	1	3
17	1	1

DOX

HC	adenomas	adencarcin
2	2	1
7	1	1
5	0	1
4	2	0
3	0	0
6	1	1
2	0	0

Frl/DOX

HC	adenomas	adencarcin
4	2	1
1	1	0
1	1	0
1	1	0
7	1	0
8	1	1
7	1	1

DMH: dimethylhydrazine
HC: hyperplastic crypts
DOX:doxorubicin
Frl: fullerenol

miocardial lesions

DHM

degenation	limf.inf.	myof.loss
2	0	1
1	1	0
1	0	0
1	1	0
1	1	0
2	0	1
1	0	1

DOX

degenation	limf.inf.	myof.loss
2	1	1
2	1	1
2	2	2
1	1	1
1	1	1
2	1	1
2	1	1

Frl/DOX

degenation	limf.inf.	myof.loss
2	0	1
1	1	0
1	0	1
1	0	0
1	0	0
2	0	0
1	0	0

BIKE analysis

DMH

MDA	GSH/GSS(	SOD	CAT
14,66	2,89	2,09	39,92
20,03	3,46	1,73	31,74
17,53	2,43	1,68	21,68
16,84	3,43	1,98	23,76
20,13	2,09	2,44	24,7
23,95	1,72	1,28	38,22
20,41		1,23	

DOX

MDA	GSH/GSS(	SOD	CAT
43,25	1,14	5,63	52,81
21,94	1,68	6,47	43,71
28,38	1,06	5,75	45,48
20,63	0,99	5,71	40,6
42,72	1,73	6,89	62,57
21,31	0,79	8,48	37,35
		9,81	38,17

Frl/DOX

MDA	GSH/GSS(	SOD	CAT
21,7	2,94	6,49	28,28
20,16	2,58	3,31	17,89
11,19	1,56	5,43	9,12
18,94	3,7	6,1	11,9
13,88	3,02	5,02	21,14
18,28	2,64	4,93	9,95
20,59	3,05		13,3
