# Supplementary material for: Cardiac autonomic modulation induced by doxorubicin in a rodent model of colorectal cancer and the influence of fullerenol pretreatment
Source: PLoS One. 2017 Jul 20;12(7):e0181632. doi: 10.1371/journal.pone.0181632 (PMC5519181; doi:10.1371/journal.pone.0181632)
Supplement: S2 Dataset — (PDF) [file pone.0181632.s002.pdf]

cancer	RR	SDNN	RMSSD	nLF	nHF	LF/HF
podg1	164,5127	1,2279	1,1981	4,044	94,064	0,046
podg2	270,3355	3,5238	5,5249	10,954	84,206	0,1301
podg3	220,5545	2,9945	7,001	18,0056	79,223	0,2273
podg4	259,8871	2,5035	4,0539	47,913	51,293	0,9004
podg5	261,2741	2,1318	3,8366	20,364	79,273	0,2569
podg6	225,6498	3,5628	5,6534	29,221	74,156	0,3341
podg7	239,473	3,8068	6,9464	24,426	71,184	0,3431

cancer+DOX	RR	SDNN	RMSSD	nLF	nHF	LF/HF
podg31	267,3632	1,8185	2,597	39,204	57,744	0,6789
podg32	322,8987	7,1787	11,3013	30,194	69,569	0,434
podg33	238,0503	11,6608	14,9429	46,227	52,868	0,8744
podg34	230,9302	8,376	9,8913	35,251	63,823	0,5523
podg35	351,892	2,8636	3,6877	36,162	63,415	0,5702
podg36	253,7784	3,3326	5,1877	40,335	57,642	0,6998
podg37	242,237	3,9091	5,3646	29,18	69,051	0,4226

cancer+DOX+FrI	RR	SDNN	RMSSD	nLF	nHF	LF/HF
podg51	267,954	5,6582	9,7917	19,421	80,077	0,2425
podg52	236,2514	4,2019	13,4913	25,01	74,198	0,3371
podg53	228,819	2,1212	2,977	20,543	77,323	0,2657
podg54	230,4653	4,2701	6,4961	10,482	88,642	0,1183
podg55	279,437	3,8569	6,0458	13,724	85,23	0,161
podg56	255,1516	2,4191	3,2996	31,846	67,303	0,4723
podg57	249,3356	2,4371	3,4933	22,57	76,697	0,2943
